# Manipulating chromatin architecture in *C. elegans*

**DOI:** 10.1186/s13072-022-00472-5

**Published:** 2022-11-29

**Authors:** John L. Carter, Colton E. Kempton, Emily D. S. Hales, Steven M. Johnson

**Affiliations:** Department of Microbiology and Molecular Biology, College of Life Sciences, Brigham Young University, Provo, UT 84602 USA

**Keywords:** Chromatin, *C. elegans*, Transgene, Nucleosomes, Nucleosome positioning, Nucleosome occupancy, Epigenetics, PRS-322, Widom 601

## Abstract

**Background:**

Nucleosome-mediated chromatin compaction has a direct effect on the accessibility of trans-acting activators and repressors to DNA targets and serves as a primary regulatory agent of genetic expression. Understanding the nature and dynamics of chromatin is fundamental to elucidating the mechanisms and factors that epigenetically regulate gene expression. Previous work has shown that there are three types of canonical sequences that strongly regulate nucleosome positioning and thus chromatin accessibility: putative nucleosome-positioning elements, putative nucleosome-repelling sequences, and homopolymeric runs of A/T. It is postulated that these elements can be used to remodel chromatin in *C. elegans*. Here we show the utility of such elements in vivo, and the extreme efficacy of a newly discovered repelling sequence, PRS-322.

**Results:**

In this work, we show that it is possible to manipulate nucleosome positioning in *C. elegans* solely using canonical and putative positioning sequences. We have not only tested previously described sequences such as the Widom 601, but also have tested additional nucleosome-positioning sequences: the Trifonov sequence, putative repelling sequence-322 (PRS-322), and various homopolymeric runs of A and T nucleotides.

**Conclusions:**

Using each of these types of putative nucleosome-positioning sequences, we demonstrate their ability to alter the nucleosome profile in *C. elegans* as evidenced by altered nucleosome occupancy and positioning in vivo. Additionally, we show the effect that PRS-322 has on nucleosome-repelling and chromatin remodeling.

**Supplementary Information:**

The online version contains supplementary material available at 10.1186/s13072-022-00472-5.

## Background

### Chromatin and gene regulation

The eukaryotic chromatin is the sum total of the DNA and DNA-interacting proteins found in nuclei. The majority of these chromatin proteins are histones that are wrapped in DNA constituting nucleosomes. Histone placement on the genome affects the accessibility of the genes and regulatory regions to trans-acting activating and repressing proteins and thus serves as a primary regulatory agent of genetic expression [1]. Understanding the nature and dynamics of chromatin is fundamental to teasing apart the mechanisms and factors that compose the field of epigenetics.

Paramount among the critical functions of chromatin are: condensing and organizing DNA, facilitating equal distribution of genetic material during cell division, and helping to regulate which genes are being expressed during development and homeostasis. The basic unit of chromatin is the nucleosome which is comprised of 147 base pairs (bp) of DNA wrapped around an octamer of histone proteins, two of each H2A, H2B, H3, and H4 [2, 3]. The intervening DNA between nucleosomes, called linker DNA, varies in length between 30 and 60 bp in human cells and varies between species, and cell types [4, 5].

At the most fundamental level, chromatin exists in two different states: euchromatin and heterochromatin. Euchromatin is loosely packed with little to no association or interaction between separate nucleosomes, whereas heterochromatin is packed densely, and nucleosomes interact to form compact structures [6]. Actively transcribed genes are typically found in areas of euchromatin [7]. Genes found in heterochromatin regions are not transcriptionally active as the DNA is packed too tightly to be accessible to transcription factors [8].

DNA sequence influences how and where nucleosomes sit across the genome [4, 9]. DNA is not a homopolymer, and each dinucleotide step along a DNA molecule has its own unique stereochemistry [10]. Consequently, DNA is anisotropic, and DNA sequences with intrinsic bending help facilitate wrapping around the histone octamer. Indeed, it has been shown that having AA/TT dinucleotides spaced every 10 bp, or every turn of the DNA helix, increases innate bending and allows the histone octamer to bind with increased affinity [10, 11]. However, longer homopolymeric runs of A/T are recalcitrant to nucleosome formation [12].

**Fig. 1:**
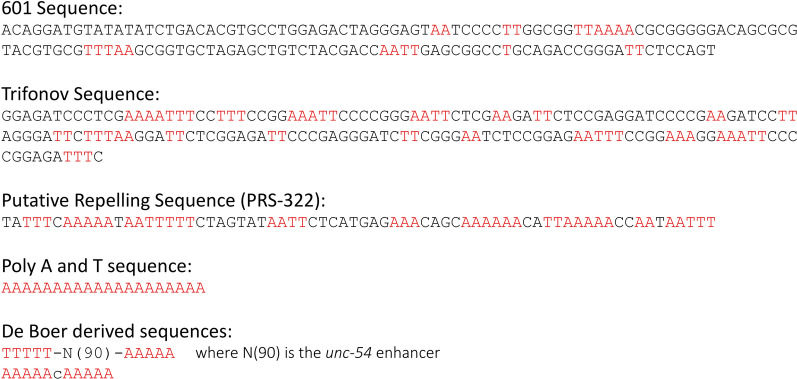
Sequences of putative positioning (601 and Trifonov), and putative repelling [PRS-322, poly(A) and poly(T) sequences and de Boer-derived sequences] DNA elements with dinucleotide or longer runs of As or Ts highlighted in red

Three different types of nucleosome-positioning sequences are tested here: (1) two putative nucleosome-positioning elements, the Widom 601 sequence [11, 13] and the Trifonov sequence [14]; (2) one novel suspected nucleosome-repelling sequence (PRS-322), and (3) different homopolymeric runs of A/T [15] (Fig. 1). The 601 sequence is a synthetically derived sequence that strongly positions nucleosomes in vitro and in vivo [11, 16] and is a standard positioning element used in the nucleosome positioning field, but its positioning properties have never been tested in *C. elegans*. The Trifonov sequence was derived by analyzing a large dataset of *C. elegans* nucleosome DNA cores isolated from whole worms [17]. Positional preferences of the different dinucleotide steps along the 147-bp nucleosome DNA cores from across the entire *C. elegans* genome were calculated, and a “bendability pattern” was derived from these calculations which would theoretically favor nucleosome formation. This resulted in a computationally optimal, putative *C. elegans* nucleosome-positioning sequence, the Trifonov sequence [14]. We have also discovered a novel positioning sequence, here named PRS-322, that has strong nucleosome-repelling properties. PRS-322 is a sequence we isolated from the *C. elegans* genome that during in vitro nucleosome reconstitution experiments repelled or impeded nucleosome formation (Additional file 1: Fig. S1). Finally, homopolymeric runs of A/T are known to be recalcitrant to nucleosome formation, have been shown to be overrepresented in nucleosome-free regions in yeast, mice, and humans [4, 18–22], and hence are putative nucleosome-repelling sequences. In yeast it has been found that poly(dA:dT) tracts affect nucleosome occupancy and gene transcription when positioned near promoters [23]. When looking at nucleosome occupancy in vivo around such elements naturally occurring in the genome, de Boer et al. (2014) found greatly different nucleosome occupancy outcomes that varied between species. These elements even had nucleosome-repelling function on opposing sides when comparing results between yeast and mammals [15]. Thus, we decided to test such de Boer-derived poly(dA:dT) sequence combinations in worms.

Here we demonstrate that using many of these positioning and repelling sequences, we can specifically modify nucleosome positioning and architecture to change the placement and occupancy of nucleosomes in living worms.

## Results

### Chromatin manipulation via addition of positioning/repelling sequences

**Fig. 2:**
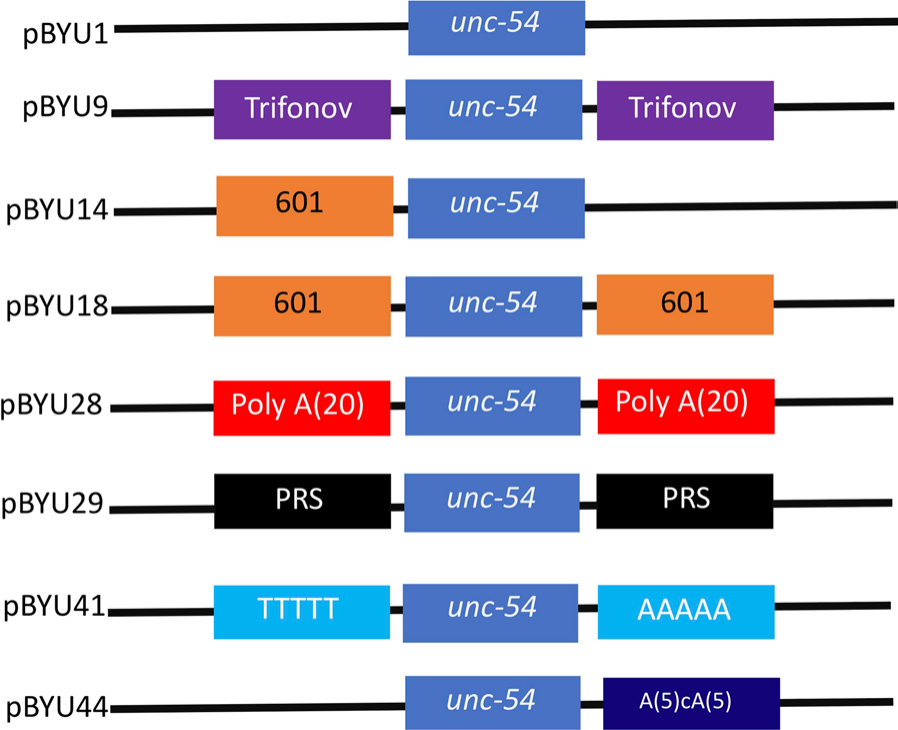
Depiction of transgene constructs. pBYU1(the control construct) with the *unc-54* minimal enhancer (blue). pBYU9, pBYU14, and pBYU18 have putative nucleosome positioning [601(orange) or Trifonov (purple)] elements around the *unc-54* minimal enhancer. pBYU28, pBYU29, pBYU41, and pBYU44 were used to test putative repelling elements; pBYU28 with 20 bp poly-A repeats (red), pBYU29 with the 70 bp PRS-322 sequence (black), pBYU41 with forward- and reverse-oriented 5 bp poly-T sequences (turquoise), and pBYU44 with an AAAAAcAAAAA sequence (dark blue) downstream of the *unc-54* minimal enhancer (blue)

We hypothesized that using nucleosome positioning and repelling sequences we could change where nucleosomes form on transgenes in living *C. elegans*. To this end, we inserted various DNA sequences into a plasmid we made, pBYU1, that was derived from the well-characterized Fire Lab plasmid pPD151.79 (see methods). The pPD151.79 plasmid has a promoter that drives green fluorescent protein (*gfp*) expression in pharyngeal muscle cells in *C. elegans*. Expression of GFP in these cells was used as an internal control to confirm maintenance of the transgene in transgenic worms. The pPD151.79 plasmid also has an *unc-54* enhancer in it. We modified the *unc-54* enhancer to be only 90 bp, to form a minimal *unc-54* enhancer. It is around this minimal enhancer that we inserted our various nucleosome-positioning elements to attempt to influence local nucleosome positions and ascertain whether gene regulatory elements affected the chromatin manipulating sequences’ ability to alter the chromatin landscape. The minimal enhancer was used because its 90 bp length would be too short to allow a nucleosome to form on the enhancer if it were flanked by positioned nucleosomes due to the inserted chromatin manipulating sequences (Fig. 2). Constructs pBYU9, pBYU14, and pBYU18 all harbor putative nucleosome-positioning sequences, while pBYU28, pBYU29, pBYU41, and pBYU44 have putative nucleosome-repelling sequences. pBYU1 serves as a control construct with neither positioning nor repelling sequences (Table 1). In constructs pBYU9, pBYU18, pBYU28, pBYU29, and pBYU41 putative nucleosome positioning/repelling sequences were inserted on both sides of the enhancer. This was to elucidate how these sequences would work in tandem with each other and with regard to the enhancer element.

**Table 1:** Transgene construct purposes and modifications

Purpose	Modification/insert	Insert location/direction		Plasmid	Worm strain
		Upstream^a^	Downstream^b^		
Control	Minimal *unc54* enhancer	–	–	pBYU1	BYU1
Positioning	601	Forward	–	pBYU14	BYU10
	601	Forward	Forward	pBYU18	BYU12
	Trifonov	Forward	Forward	pBYU9	BYU6
Repelling	A(5)-C-A(5)	–	Reverse	pBYU44	BYU19
	T(5)	Forward	Reverse	pBYU41	BYU15
	A(20)	Forward	Forward	pBYU28	BYU14
	PRS-322	Forward	Reverse	pBYU29	BYU13

^a^Inserted in the HindII site upstream of the *unc-54* minimal enhancer

^b^Inserted in the NheI site downstream of the *unc-54* minimal enhancer

### Confirmation of changes in transgene nucleosome occupancy

**Fig. 3:**
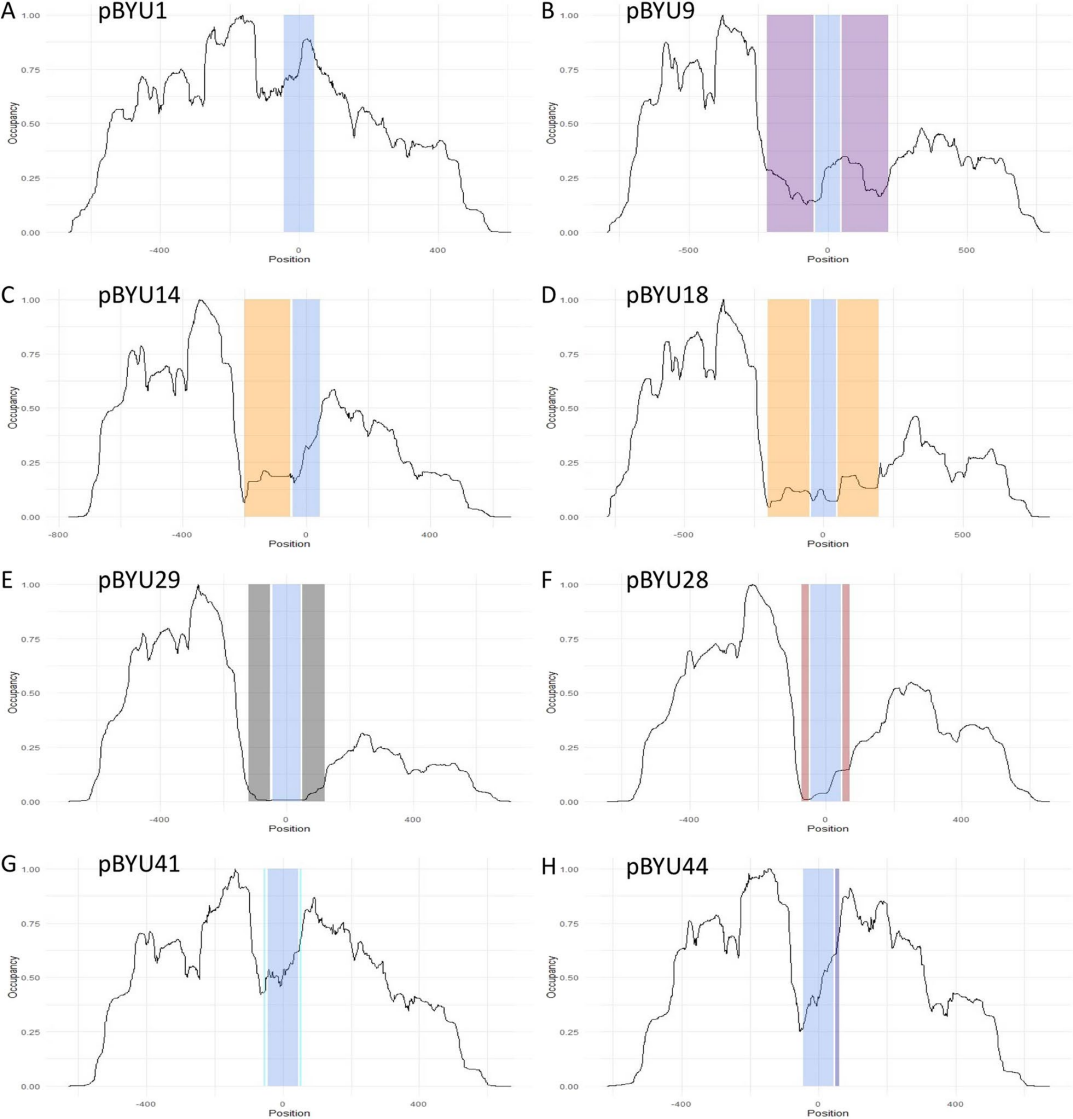
Nucleosome occupancy, the overall number of nucleosomes bound to DNA at a given locus. In all constructs the *unc-54* minimal enhancer locus is present and highlighted in blue with the enhancer center designated as the origin and the x-axis numbering in base pairs. The y-axis is the normalized nucleosome occupancy. **A** pBYU1, with only the *unc-54* minimal enhancer locus. **b** pBYU9 with the Trifonov sequence loci highlighted in purple. **C** pBYU14 with the 601 sequence locus in orange. **D** pBYU18 with the two 601 sequence loci in orange. **E** pBYU29 with the two 70-bp PRS-322 loci in gray. **F** pBYU28 with the two 20-bp poly-A runs highlighted in red. **G** pBYU41 with the forward and reverse 5-bp poly-T runs in light blue. **H** pBYU44 with the single AAAAAcAAAAA sequence locus in dark blue

**Fig. 4:**
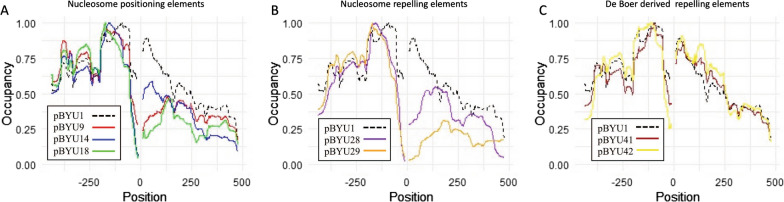
Comparative nucleosome occupancy of regions flanking the *unc-54* enhancer and inserted elements. **A** Nucleosome occupancy for the flanking regions for pBYU1 (dashed black) compared to the flanking regions from transgenes with positioning elements: pBYU9 (red), pBYU14 (blue), pBYU18 (green). **B** Nucleosome occupancy for the flanking regions for pBYU1 (dashed black) compared to the flanking regions from transgenes with repelling elements: pBYU28 (purple), pBYU29 (orange). **C** Nucleosome occupancy for the flanking regions for pBYU1 (dashed black) compared to the flanking regions from transgenes with repelling elements derived from the de Boer analysis: pBYU41 (brown), pBYU44 (yellow). For all three graphs, the *unc-54* enhancer and the inserted positioning/repelling sequences have been removed with position 0 being where those elements would have been located. Negative x-axis values are the number of base pairs upstream, and positive *X*-axis values are the number of base pairs downstream of these elements

**Fig. 5:**
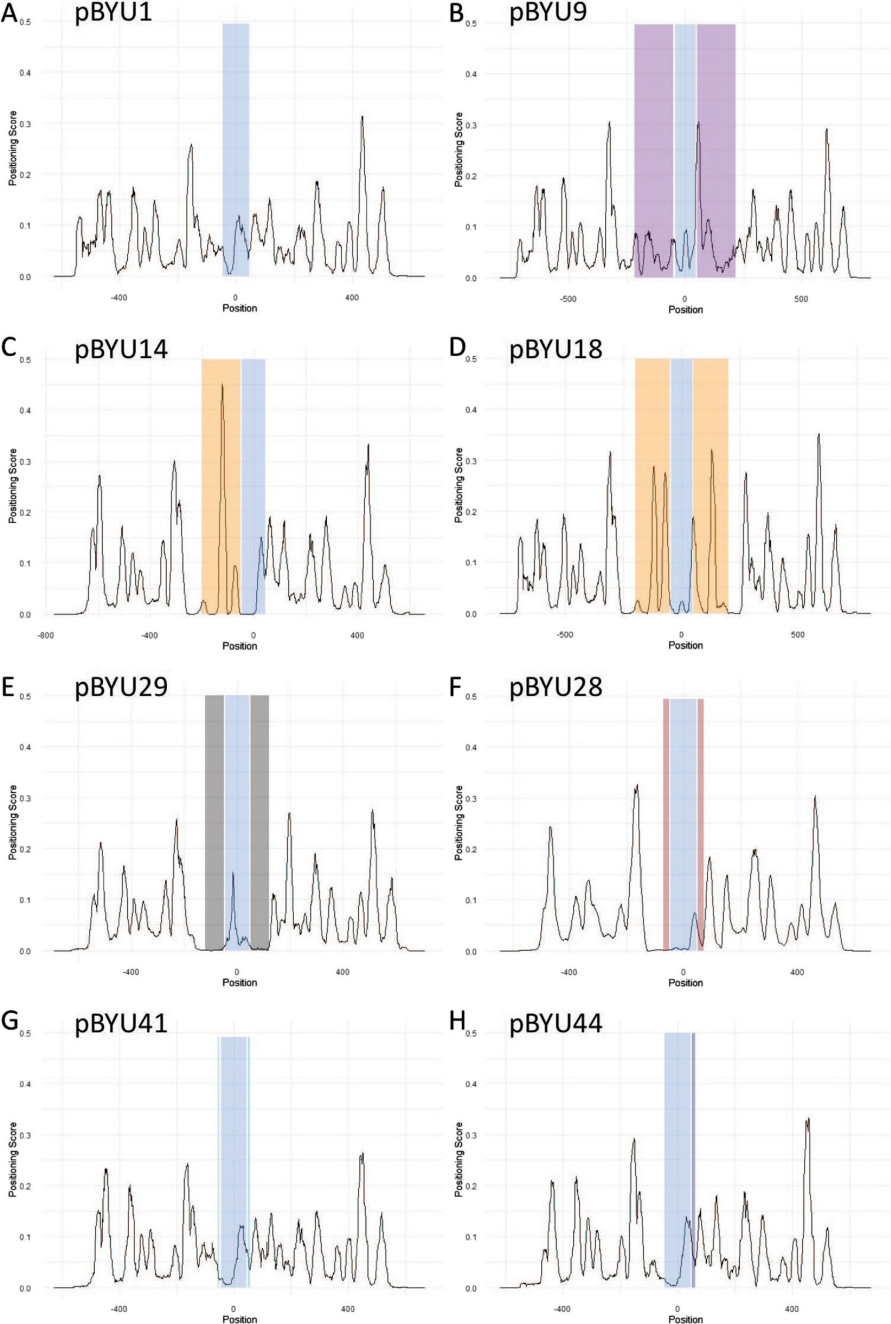
Nucleosome positioning, the percent of nucleosomes (dyads) positioned within a 21-bp window surrounding a locus compared to all the nucleosomes (dyads) within the 301-bp window surrounding that same locus. As in Fig. 3, in all constructs, the *unc-54* minimal enhancer locus is present and highlighted in blue with the enhancer center designated as the origin and the x-axis numbering in base pairs. The y-axis is the percent nucleosome positioning score represented as a decimal. **A** pBYU1, with only the *unc-54* minimal enhancer locus. **B** pBYU9 with the Trifonov sequence loci highlighted in purple. **C** pBYU14 with the 601 sequence locus in orange. **D** pBYU18 with the two 601 sequence loci in orange. **E** pBYU29 with the two 70 bp PRS-322 loci in gray. **F** pBYU28 with the two 20 bp poly-A runs highlighted in red. **G** pBYU41 with the forward and reverse 5 bp poly-T runs in light blue. **H** pBYU44 with the single AAAAAcAAAAA sequence locus in dark blue

We expected that the above modifications made to our transgenes would result in modified nucleosome positions on the transgenes in vivo as hypothesized. When injected into worms, these transgenes form repetitive extra-chromosomal arrays that are transmitted from generation to generation in *C. elegans*. Thus, we isolated transgene nucleosome DNA cores from these in vivo arrays by MNase digestion and selective hybridization [9, 16, 17]. Transgene nucleosome occupancy and positioning were assessed through next generation sequencing of the isolated DNA cores. We observed drastic changes in nucleosome occupancy and positioning on our transgenes in vivo (Figs. 3, 4, and 5).

Nucleosome occupancy is defined as the overall number of nucleosomes bound to DNA at a given locus. As shown in Fig. 3, in vivo nucleosome occupancy levels decreased at the *unc-54* minimal enhancer locus (*unc-54* locus) on transgenes with the added positioning elements (pBYU9, pBYU14, and pBYU18), when compared to pBYU1. The same decrease was seen in all transgenes with repelling sequences (pBYU28, pBYU29, pBYU41, and pBYU44). However, this decrease was less pronounced on transgenes pBYU41 and pBYU44. The decrease was much more pronounced on transgenes pBYU28 and pBYU29 in transgenic lines, with virtually no nucleosomes occupancy at the *unc-54* locus in pBYU29 (Fig. 3E) which harbored our novel PRS-322 sequence. The amount of nucleosome occupancy on the *unc-54* enhancer was calculated for each construct and compared to the control (pBYU1). Table 2 shows the total normalized occupancy and the total normalized occupancy divided by the normalized occupancy of pBYU1. We see that all of our modifications resulted in reduced nucleosome occupancy, but that pBYU 29 (the PRS) virtually eliminated nucleosome occupancy by reducing occupancy on the *unc-54* enhancer by 99%.

**Table 2:** Normalized occupancy at the *unc-54* loci

	pBYU1	pBYU9	pBYU14	pBYU18	pBYU28	pBYU29	pBYU41	pBYU44
Total occupancy	62.73911	24.08857	26.62738	8.140605	6.199199	0.677021	48.74391	41.2828
Normalized to pBYU1	1	0.383948	0.424414	0.129753	0.098809	0.010791	0.77693	0.658007

Beyond nucleosome occupancy on the *unc-54* enhancer, we wanted to see the effect of the introduced DNA elements on nucleosome occupancy both upstream and downstream of the inserted sequences. Occupancy in the flanking regions around the positioning/repelling/*unc-54* loci were compared and quantified for each construct (Fig. 4). While nucleosome occupancy upstream of the added DNA sequences is very similar for all the constructs, occupancy downstream of the inserted sequences was altered for most of the constructs. Despite the upstream nucleosome occupancy pattern being generally the same as the control, we did see a steep drop in occupancy at position − 50, from 0.65 in pBYU1 to 0.1 in both pBYU28 and pBYU29. A similar upstream drop is seen in pBYU14 and pBYU18, with more moderate drops at pBYU9, pBYU41 and pBYU42.

Downstream nucleosome occupancy was much more affected. Specifically, in the case of the nucleosome-positioning elements (pBYU14, pBYU18 and pBYU9), all have a similar profile downstream of the elements: the first downstream peak is reduced from 0.9 in the control to 0.6 in the pBYU14 and even more reduced down to 0.25 and 0.4 for pBYU18, and pBYU9, respectively, at position  + 75. At position  + 150 we see a valley that is nearly identical across all three constructs and control. Downstream of that position the general profile is the same, but reduced by approximately 10 to 50 percent across the three constructs compared to the control. Two of the repelling sequences (pBYU28 and pBYU29) have reduced downstream occupancy with similar profiles as well. Downstream, at position  + 50 we see a complete loss of the first downstream peak with a drop from 0.8 in the control to 0.3 in pBYU28 and 0.1 in pBYU29. We also see the downstream occupancy in these constructs peak at position  + 135 at 0.55 in pBYU28 and position  + 185 at 0.3 in pBYU29. This is in juxtaposition to the valley in the control over the same positions. The other two repelling sequences (pBYU42 and pBYU44) had minimal downstream occupancy changes, with a profile similar to the unmodified pBYU1. Interestingly there is a peak at position + 125 in both pBYU42 and pBYU44 that is absent in the control, and in these two constructs we see a decrease in the relative depth of valley at position + 135 relative to the control.

### Confirmation of changes in transgene nucleosome positioning

Nucleosome positioning is the number of nucleosome dyads within 20 bp of a locus compared to the number of nucleosome dyads within 300 bp (150 bp on either side) of said locus [9]. It is interesting to note that in our transgenes, while the overall nucleosome occupancy was not high at the Trifonov or 601 sequences (Fig. 3B, C, D), the nucleosome positioning at the 601 sequences, particularly in pBYU14, was very strong with a positioning score of 0.450655359 at the center of the 601 sequences for this construct (Fig. 5C). A positioning score of 1 would indicate perfect (100%) nucleosome positioning. The 601 sequences in pBYU18 have strong positioning scores of 0.285016287 and 0.302695, upstream and downstream of the *unc-54* locus, respectively (Table 3, Fig. 5D). Thus, all the elements tested affect nucleosome formation and/or location, with the 601 sequence being particularly effective at positioning nucleosomes in *C. elegans *in vivo. The Trifonov sequence, on the other hand, demonstrated virtually no nucleosome-positioning ability in vivo with all positioning scores being less than 0.035, about an order of magnitude lower that the 601 positioning scores (Table 3).

**Table 3:** In vivo positioning scores for 601 and Trifonov elements

**Strains (constructs), Elements and Positioning Scores**	
**BYU6 (pBYU9)**	
Upstream Trifonov^a^	Downstream Trifonov^a^
Position/positioning score	Position/Positioning score
− 132/0.033762058	131/0.02213868
− 131/0.035331906	132/0.019950125
**BYU10 (pBYU14)**	
Upstream 601	
Position/positioning score	
− 122/0.450655359	
**BYU12 (pBYU18)**	
Upstream 601	Downstream 601
Position/positioning score	Position/positioning score
− 122/0.285016287	132/0.302695

^a^The Trifonov sequence is 166 bp with no true center, thus the scores are calculated at both of the two positions flanking the center

## Discussion

Heterochromatic regions of the genome are not accessible to other proteins such as transcription factors. Therefore, genes inside heterochromatin are not expressed. We postulated that the addition of nucleosome attracting, or repelling DNA sequences could be used to maintain an open chromatin state. We demonstrated the efficacy of such chromatin remodeling in vivo in *C. elegans*.

The overall nucleosome occupancy of the unmodified *unc-54* minimal locus in pBYU1 is high and is akin to a heterochromatic state. The various DNA sequences tested here have all modified the nucleosome occupancy and positioning profiles at this locus as we expected. While the attracting sequences (Trifonov and 601) did not amass high levels of nucleosome occupancy, they have altered the overall nucleosome landscape and greatly reduced occupancy at the *unc-54* locus. Additionally, the repelling sequences in pBYU28 and pBYU29 greatly reduced the occupancy of nucleosomes at the *unc-54* locus, with the pBYU29 PRS-322 repelling sequence bringing the occupancy down drastically, producing essentially a nucleosome-free *unc-54* minimal enhancer.

Repelling sequences pBYU41 and pBYU44 were derived from the data in the de Boer paper *Poly-dA:dT Tracts Form an *In Vivo* Nucleosomal Turnstile* [15]. Therefore, we desired to compare our results to those from the de Boer study. De Boer looked at in vivo nucleosome occupancy around naturally occurring poly-dA:dT tracks across the yeast, murine, and human genomes. Looking at nucleosome occupancy on and around runs of poly-T(5)… poly-A(5) in yeast, where the spacing between the TTTTT and AAAAA varies between 0 and ~ 400 bp, de Boer concluded that poly-T(5)…poly-A(5) correlates with nucleosome depletion in between the homopolymeric elements. This was not the case in mammals, with the depletion being more diffuse. In *C. elegans*, with our pBYU41 construct which has poly-T(5)-*unc-54* enhancer-poly-A(5), we observed that this configuration of homopolymeric runs behaves like the de Boer yeast data, and not only correlates with nucleosome depletion as in de Boer, but is the cause of the depletion in our transgene. Furthermore, our pBYU44 construct which has a homopolymeric run of AAAAAcAAAAA showed an increased depletion of nucleosomes from this element out to about 150 bp upstream of it across the *unc-54* enhancer. De Boer saw this same result in yeast; however, in mammals the opposite was true with the depletion of nucleosomes appearing downstream of the element. Thus, in our experiments, both of the homopolymeric motifs that were derived from the de Boer analysis recapitulated what was observed in the yeast genome and were the actual cause of the observed nucleosome occupancy depletions in *C. elegans*.

While all of our constructs reduced occupancy on the *unc-54* locus, they had additional effects on nucleosome occupancy in the regions flanking this locus. In addition to the upstream effects of the de Boer-derived constructs mentioned above, four of the other five constructs (pBYU14, pBYU18, pBYU28 and pBYU29) caused the upstream nucleosome occupancy to decreased by almost an order of magnitude at position  + 50 compared to the control. At position  + 50, pBYU9 had a depletion similar to those of pBYU41 and pBYU42. While upstream occupancy changes were limited to a 50 bp effect, downstream nucleosome occupancy changes are striking in that the distance of the changes in nucleosome occupancy extended as far as 500 bp from the modifications. With the exception of the subtle downstream nucleosome occupancy changes from the de Boer derived constructs, the other five constructs caused very large decreases in nucleosome occupancy downstream of the modified *unc-54* locs. In the case of our positioning elements (pBYU9, pBYU14 and pBYU18), these reductions did not change the pattern of the downstream occupancy significantly, but just the amplitude of the occupancy; whereas, pBYU28 and pBYU29 caused drastic changes in both pattern and amplitude of downstream nucleosome occupancy, suggesting a much larger effect on the local chromatin architecture with major, extended remodeling.

While the 601 sequence did not increase the occupancy of nucleosomes in our experiments, it did position nucleosomes with significant precision (Fig. 5C and D). While the quantity of nucleosomes positioned at the 601 loci was decreased (Fig. 3C and D), the quality of positioning of those nucleosomes was high (Table 3). We see this best in the pBYU14 data (Fig. 5C), which produced a very strong positioning signal. Thus, the 601 sequence does cause nucleosome positioning in *C. elegans,* whereas the Trifonov sequence has no such effect.

The decrease in nucleosome occupancy on the 601 loci seen in our experiments is not without precedent. When the 601 sequence was tested in vivo by Gracey et al. [16], they observed high occupancy and strong positioning in mice 3 days after introducing the 601-harboring plasmid, but both the occupancy and positioning had disappeared after 6 weeks. Interestingly, in these experiments, the occupancy and positioning were high when expression of their transgene was high, and the loss of both occupancy and positioning coincided with silencing of the transgene they were using. Thus, long-term maintenance of nucleosome occupancy on positioning elements may not be expected, and our lack of nucleosome occupancy on our 601 constructs is similar to the results seen by Gracey et al. [16], and may be due to the fact that in order to have a sufficient mass of worms, our transgenic *C. elegans* were propagated for multiple generations. The retention of the high degree of nucleosome positioning on 601 in our experiments might be more what one might expect despite not having seen the same effect in the Gracey study.

## Conclusions

Here we have demonstrated that the 601 sequence provides a strong positioning signal for long-term nucleosomes positioning in *C. elegans*. Additionally, we tested a new nucleosome-repelling element, PRS-322, and demonstrated that it repels nucleosomes in vivo better than the homopolymeric runs of A/T that we tested, with near 100% repelling activity. Additionally, the PRS-322 element causes extensive chromatin remodeling and nucleosome depletion, especially downstream of the element in vivo. With further in vitro and in vivo testing in other organisms and systems, we anticipate that the PRS-322 elements, acting with opposing function to 601, might be of great use in the chromatin field, and as 601 is the gold standard for nucleosome positioning, PRS-322 could become the standard for nucleosome-repelling elements.

## Methods

### Plasmid constructs

**Fig. 6:**
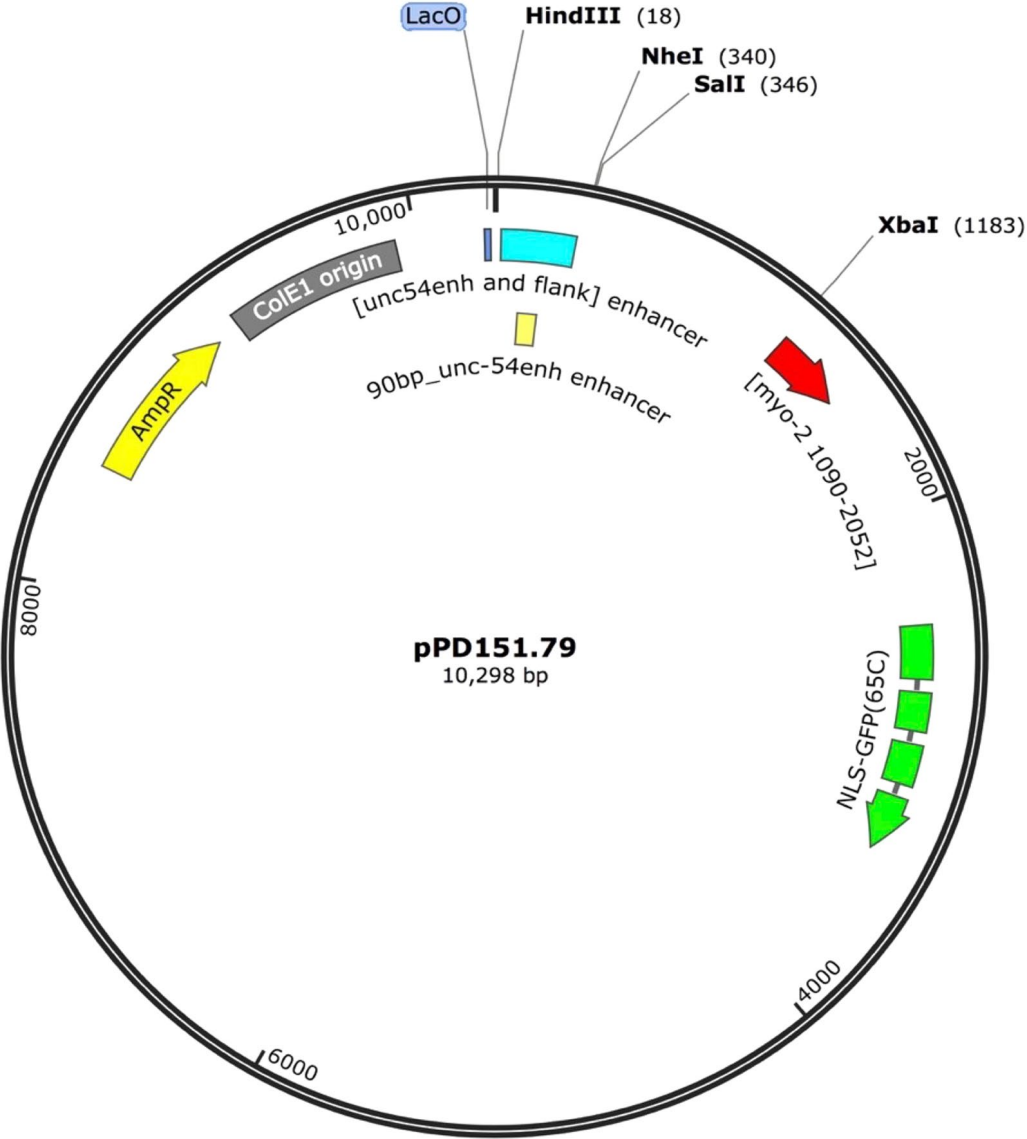
Plasmid map of pPD151.79

The construct plasmids, pBYU1 and pBYU2 were made from the pPD151.79 plasmid backbone which contains the *unc-54* enhancer upstream of the *myo-2* promoter, which promotes a nuclear-localized GFP::lacZ fusion protein (Fig. 6). pBYU1 was created by cutting pPD151.79 with HindIII and NheI followed by inserting by ligation the minimal *unc-54* enhancer. The minimal *unc-54* enhancer was generated by annealing primers SJ-BYU-1 and SJ-BYU-2 together and extending them out to full length by annealing PCR at 51 °C for 25 cycles followed by TOPO cloning (Invitrogen) of the product. After sequencing to confirm the correct sequence, the TOPO-minimal enhancer plasmid was digested with HindIII and NheI, and the minimal enhancer was isolated on a 2% agarose gel and by gel extraction. pBYU2 was created by cutting pPD151.79 with HindIII and NheI, followed by end blunting and religating the vector without an insert. pPD151.79 and pBYU1 were cut with both SalI and XbaI, the resulting vectors isolated and blunted and then re-ligated to make pBYU3 and pBYU4, respectively. The remaining plasmids were made from the pBYU1 backbone. pBYU9 was made by cutting pBYU1 first at the *HindIII* site followed by calf intestinal phosphatase (CIP) treatment and inserting a forward-oriented Trifonov sequence and then subsequentially cutting this resulting plasmid at the *NheI* site followed by CIP treatment and inserting a second forward-oriented Trifonov sequence. The Trifonov sequences were generated via PCR with CK-SJ-5 and CK-SJ-6 for the HindIII-Trifonov sequence and primers CK-SJ-7 and CK-SJ-8 for the NheI-Trifonov sequence using a plasmid that contained the Trifonov sequence as template. pBYU18 followed the same method as pBYU9 but with forward-oriented 601 sequences in the place of Trifonov sequences. The 601 sequences were generated by PCR amplification with a plasmid which harbored the 601 sequence (a gift from the Narlikar lab) with primers HindIII601 F, HindIII601 R, and NheI601 F, NheI601 R, respectively. pBYU14 was made in the same fashion but without inserting a 601 sequence at the *NheI* site. Plasmids pBYU28 and pBYU41 were made by annealing primer pairs together to make the appropriate inserts and then sequentially inserting the upstream or downstream sequences into the pBYU1 plasmid as described above. For pBYU28 the annealed upstream primers were H_F_polyT, and H_R_polyT, and the annealed downstream primers were N_F_polyT, and N_R_polyT. For pBYU41 the annealed upstream primers were HindIII_5a and HindIII_5t, and the annealed downstream primers were NheI_5t and NheI_5a. For pBYU29, primers 70F_H_pSJ322 and 70R_H_pSJ322 (upstream insert), and 70F_N_pSJ322 and 70R_N_pSJ322 (downstream insert) were used to amplify the 70 bp PRS-322 from plasmid pSJ322. The inserts were then TOPO cloned as described for pBYU1, and then sequenced, digested and sequentially ligated into the *HindIII* and *NheI* cut sites as described for pBYU9. Lastly, for pBYU44, primers 0SMJ023 and oSMJ024 were annealed to make the insert as described for pBYU28 and ligated into the downstream *NheI* site of the pBYU1 plasmid. All construct sequences were confirmed by Sanger sequencing, and all primers used in their construction are listed in Table 4.

**Table 4:** Sequences of primers used in plasmid construction and biotinylated DNA probe generation

Primer name	Primer sequence
SJ-BYU-1	AAGCTTATCCCATTCTCTCATCAATTGAGTGGGATGAGGCTATCTCTGCCTCTCTTCTGA
SJ-BYU-2	GCTAGCGTCATCCACAGTGTAATGTAAGATGGTTCAGAGATTCAGAAGAGAGGCAGAGAT
CK-SJ-5	ACTAGCAAGCTTGGAGATCCCTCGAAAATTTC
CK-SJ-6	GTGCTCAAGCTTGGAAATCTCCGGGGAATTTC
CK-SJ-7	ACTAGCGCTAGCGGAGATCCCTCGAAAATTTC
CK-SJ-8	GTGCTCGCTAGCGGAAATCTCCGGGGAATTTC
HindIII601 F	AAGCTTACAGGATGTATATATCTGACACG
HindIII601 R	AAGCTTACTGGAGAATCCCGGTCTGC
NheI601 F	GCTAGCACAGGATGTATATATCTGACACG
NheI601 R	GCTAGCACTGGAGAATCCCGGTCTGC
NheI_5t	/5Phos/CTAGCTTTTTG
NheI_5a	/5Phos/CTAGCAAAAAG
HindIII_5t	/5Phos/AGCTTTTTTTA
HindIII_5a	/5Phos/AGCTTAAAAAA
N_R_polyT	/5Phos/CTAGCAAAAAAAAAAAAAAAAAAAAG
N_F_polyT	/5Phos/CTAGCTTTTTTTTTTTTTTTTTTTTG
H_R_polyT	/5Phos/AGCTTAAAAAAAAAAAAAAAAAAAAA
H_F_polyT	/5Phos/AGCTTTTTTTTTTTTTTTTTTTTTTA
70R_N_pSJ322	GCTAGCAAATTATTGGTTTTTAATGTTTTTTGCTG
70F_N_pSJ322	GCTAGCTATTTCAAAAATAATTTTTCTAGTATAATTC
70R_H_pSJ322	AAGCTTAAATTATTGGTTTTTAATGTTTTTTGCTG
70F_H_pSJ322	AAGCTTTATTTCAAAAATAATTTTTCTAGTATAATTC
0SMJ023	/5Phos/CTAGCTTTTTGTTTTTG
oSMJ024	/5Phos/CTAGCAAAAACAAAAAG
oSMJ118	ATGGAAAAACGCCAGCAACG
oSMJ119_5Biotin	TATGAGGACGGTATACATTCG

### Generation of transgenic worms

All worm strains were generated following standard procedures for *C. elegans* microinjection [24, 25]. Each strain was injected with 50 ng/ul of construct plasmid and 50 ng/ul of *rol6(su1006)* pRF4 plasmid as a co-injection marker. Table 1 shows which plasmids were injected to make each transgenic worm strain. Multiple individual transgenic lines (a minimum of three) for each strain were generated to control for the inherent variation between extra-chromosomal arrays.

### In vivo transgene nucleosome isolation

To isolate the nucleosomes that formed in vivo on the various transgenes, transgenic worms were proliferated on nematode growth media (NGM) plates with a lawn of *E. coli* strain OP50. Since transgenic worms with non-integrated transgenes are mosaics and do not throw 100% transgenic progeny, we enriched our transgenic worm populations as described by Carter, et al. [26]. Enriched transgenic worm populations were flash frozen into pellets with liquid nitrogen and stored at − 80 °C.

Frozen worm pellets were crushed under liquid nitrogen using a mortar and pestle, and once the samples were thawed on ice, mononucleosome DNA cores were isolated as previously described [9, 17], with MNase digestion for 15 min at 16 °C with various MNase concentrations (0.5, 2.5 and 12.5 units/µL) for each sample to achieve optimal chromatin digestion and mononucleosome core DNA isolation. All experiments were conducted on whole worms and therefore lack tissue specificity.

### Illumina library prep

Mononucleosome core DNA samples were made into sequencing libraries according to the Illumina Library prep protocol (Illumina 1003806 Rev. A) with mononucleosome DNAs from each transgenic line being indexed with unique barcodes for multiplex sequencing.

### Selective hybridization and sequencing

For each library, biotinylated DNA probes that cover the entire transgene sequence of interest plus a portion of the DNA sequence flanking the target were generated via PCR of the respective plasmids using a non-biotinylated forward primer (oSMJ118) and a biotinylated reverse primer (oSMJ119_5Biotin) (Table 4).

The probes and libraries were denatured, and single strand mononucleosome core DNA was hybridized to the probe and pulled down with magnetic streptavidin-coated beads from Promega. The hybridization was performed as described in Gracey et al. [16]. The mononucleosome core DNA was then amplified via 12 cycles of PCR and sequenced following standard Illumina 25-bp paired-end sequencing [27]. Total sequencing coverage of the transgene was calculated based on the extra-chromosomal array having between 70 and 300 copies of the transgene and is presented in Table 5.

**Table 5:** Total reads, reads mapped to probe, and estimated coverage for each construct

Construct	Total reads	Reads mapped to probe	% Mapped to probe	Estimated coverage
pBYU1	262,562	107,151	40.81	52.5–197x
pBYU3	67,590	27,841	41.19	15–64.5x
pBYU4	180,062	77,076	42.81	49–210x
pBYU9	276,800	88,548	31.99	43–162.5x
pBYU14	1,787,380	1,083,867	60.64	531–1992x
pBYU18	209,126	91,701	43.85	45–168.5x
pBYU28	2,806,442	2,414,382	86.03	1207–4526x
pBYU29	871,718	225,164	25.83	110–414x
pBYU41	4,927,254	2,334,040	47.37	1144–4289x
pBYU44	2,406,188	1,033,938	42.97	507–1900x

### Nucleosome occupancy and positioning calculation

Fastq files produced from Illumina sequencing were aligned to the probe sequence with Burrows-Wheeler Aligner (BWA) [28]. BWA was chosen after extensive testing with simulated data sets made with ART [29]. BWA was used with default parameters except for limiting fragment length to 200 bp. The fragment length limitation insured that the reads were aligned more accurately in data sets with repeats in the constructs. Sequence Alignment/Map (SAM) files were then run through a custom python/R pipeline to calculate nucleosome occupancy and positioning. Only fragments that fell between 136 and 158 bp were used in the analysis. Occupancy was calculated by finding the dyad of each paired-end read and extrapolating 73 bp out in each direction to the canonical 147 bp nucleosome size. Positioning was calculated by dividing the number of dyads within 20 bp of each position (10 bp on each side) by the number of dyads within 300 bp (150 bp on each side) of the same position. These data were then plotted, and graphs were generated using R. Nucleosome occupancy for the *unc-54* element was calculated by adding the occupancy value for each position in the *unc-54* enhancer area, resulting in total occupancy (Table 2). Further analysis was achieved by dividing the total occupancy of each condition by that of the control pBYU1.  Initial positioning data for the hyperperiodic PRS-322 fragment [30] can be found in Fig. S1 and Additional file 2, which shows that the in vitro nucleosome formation did not suffer from end bias [31] and describes how the experiments were performed [32].

## Supplementary Information

**Additional file 1. Figure S1 ***In vitro* nucleosome reconstitution on DNA with PRS-322. Nucleosomes were reconstituted *in vitro* using salt dialysis on a 220bp (A) or a 600bp (B) fragment of DNA harboring the 70-bp PRS-322 element (highlighted in yellow). In both A and B the PRS-322 DNA and the flanking, non-highlighted DNA sequences are from the *C. elegans* genome, while the remaining sequences (highlighted in grey) are from the cloning vector. Below the full-length 220bp fragment sequence are the sequences from 27 aligned *in vitro* reconstituted nucleosome DNA cores derived from the 220bp fragment (A). Below the full-length 600bp fragment sequence are the sequences from 26 aligned *in vitro* reconstituted nucleosome DNA cores from the 600bp fragment (B). All sequence reads use the same highlighting scheme as the full-length DNA fragments from which they were derived. In both A and B, red arrows indicate nucleosomes that are potentially positioned due to end-bias.

**Additional file 2.  Positioning data and methods for Additional file 1. Figure S1**

## Data Availability

All scripts and raw data are available at https://github.com/Johnson-Lab-BYU/Nucleosome_Occupancy-Positioning.

## Abbreviations

PRS: Putative nucleosome-repelling sequence
CIP: Calf intestinal phosphatase
NGM: Nematode growth media
BWA: Burrows-Wheeler Aligner
SAM: Sequence Alignment/Map
