# Supplementary figures and images for: Manipulating chromatin architecture in C. elegans
Source: Epigenetics Chromatin. 2022 Nov 29;15:38. doi: 10.1186/s13072-022-00472-5 (PMC9706983; doi:10.1186/s13072-022-00472-5)

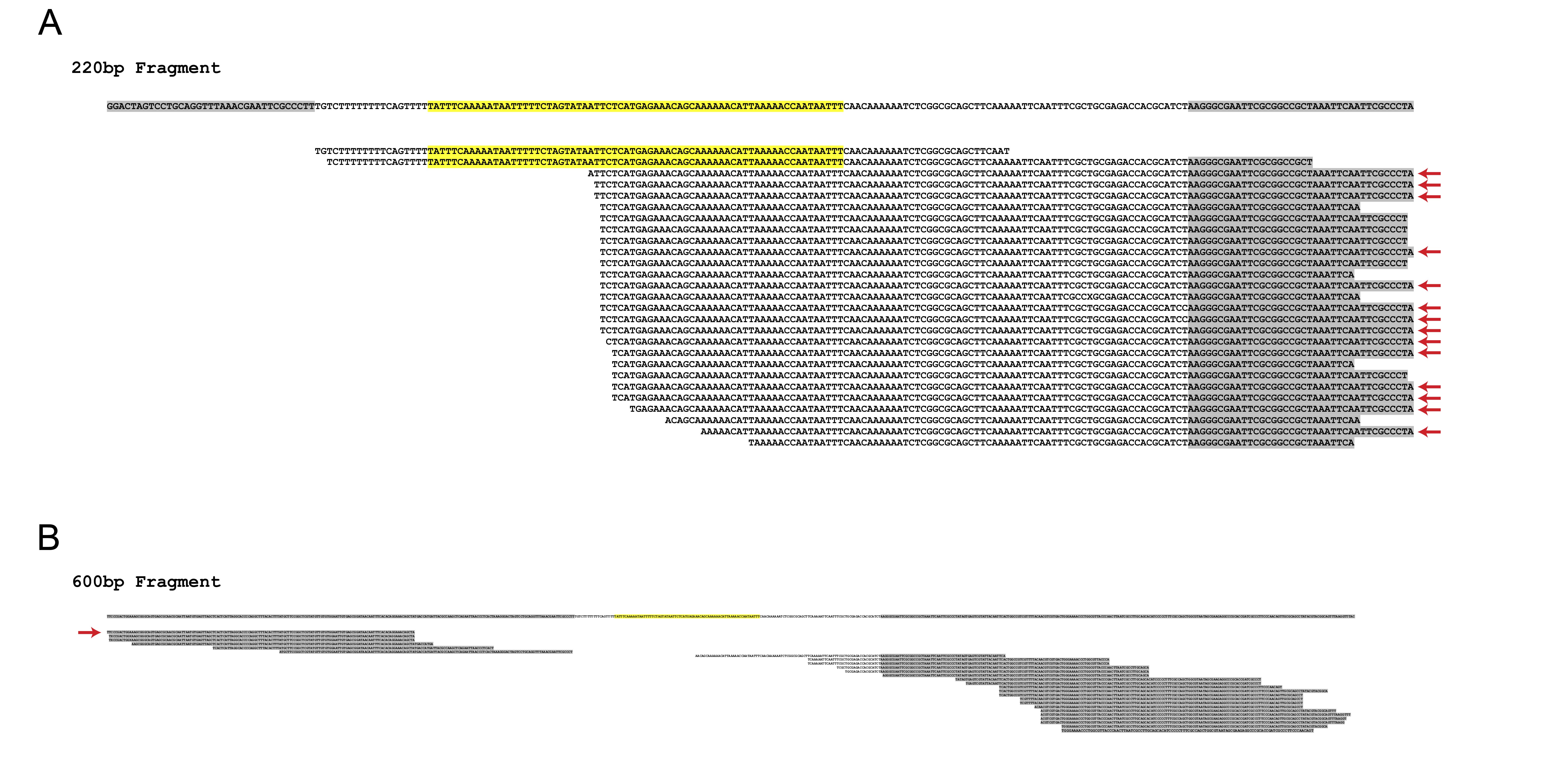

Supplement: Supplementary file 1 — Additional file 1. Figure S1 In vitro nucleosome reconstitution on DNA with PRS-322. Nucleosomes were reconstituted in vitro using salt dialysis on a 220bp (A) or a 600bp (B) fragment of DNA harboring the 70-bp PRS-322 element (highlighted in yellow). In both A and B the PRS-322 DNA and the flanking, non-highlighted DNA sequences are from the C. elegans genome, while the remaining sequences (highlighted in grey) are from the cloning vector. Below the full-length 220bp fragment sequence are the sequences from 27 aligned in vitro reconstituted nucleosome DNA cores derived from the 220bp fragment (A). Below the full-length 600bp fragment sequence are the sequences from 26 aligned in vitro reconstituted nucleosome DNA cores from the 600bp fragment (B). All sequence reads use the same highlighting scheme as the full-length DNA fragments from which they were derived. In both A and B, red arrows indicate nucleosomes that are potentially positioned due to end-bias. [file 13072_2022_472_MOESM1_ESM.jpg]
