# Supplementary material for: Manipulating chromatin architecture in C. elegans
Source: Epigenetics Chromatin. 2022 Nov 29;15:38. doi: 10.1186/s13072-022-00472-5 (PMC9706983; doi:10.1186/s13072-022-00472-5)
Supplement: Supplementary file 2 — Additional file 2. Positioning data and methods for Additional file 1. Figure S1 [file 13072_2022_472_MOESM2_ESM.docx]

**ADDITIONAL FIGURE DATA**

The nucleosome repelling ability of PRS-322 was first observed when *in vitro* nucleosome reconstitution experiments revealed that nucleosomes almost exclusively formed only on one end of a cloned *C. elegans* genomic DNA fragment which had been shown to have intrinsic curvature and to be hyperperiodic for AA/TT dinucleotides [30] (Additional Figure 1A). This fragment was only 220 base pairs in length, and a majority of the sequenced nucleosomes (14 out of 27) formed flush to the 3’ end of the DNA fragment suggesting potential end-bias effects in the experiment [31]. The concern over potential end-bias being the cause of lack of nucleosome formation on the PRS-33 sequence was allayed by the same nucleosome reconstitution experiment being performed on a 600 base pair fragment harboring the same *C. elegans* genomic DNA, which revealed no such end bias, as only one of the 26 sequence nucleosome cores was found to coincide with the end of the 600bp DNA fragment, while replicating the lack of nucleosome formation on the PRS-322 sequence (Additional Figure 1B). Three other nucleosome cores were one to two base pairs from the end of the DNA fragment, but as shown by Bates et al, such cores are most likely not at those positions due to end-bias [31]. Thus, the exclusion of nucleosomes on the 5’-end of the 220bp DNA fragment and the almost complete exclusion of nucleosome formation on the PRS-322 sequence on the 600bp DNA fragment, suggested that the PRS-322 sequence might be a potent nucleosome repelling element.

**ADDITIONAL DATA METHODS**

**PRS-322 *in vitro* nucleosomes reconstitutions**

Two separate PRS-322 harboring DNA fragments were generated using PCR from the pSJ322 plasmid that contained 147 base pairs of *C. elegans* genomic DNA from chromosome IV:11,695,705- 11,695, 851 (positions based on the Feb. 2013 assembly WBcel235/ce11) cloned into the pCR4Blunt-TOPO plasmid (Life Technologies). A 220bp fragment was made using the following primers: 5’-GGACTAGTCCTGCAGGTTTAAACGAA-3’ and 5’-TAGGGCGAATTGAATTTAGCGGCCGC-3’; and a 600bp fragment was made using the following primers: 5’-TTCCCGACTGGAAAGCGGGCAGTGAG-3’ and 5’-GTAAACCTTAAACTGCCGTACGTATA-3’ (Additional Figure 1). These PRS-322 harboring DNA fragments were used in separate *in vitro* nucleosome reconstitutions using salt dialysis, and nucleosome core DNA isolation and recovery was performed using MNase digestion as described in Kempton et al (2014) [32]. Nucleosome core DNAs were end-polished, cloned and sequenced as described in Johnson et al 2006 [17]. All sequencing of PRS-322 nucleosome core DNA was done with Sanger sequencing.

**ADDITIONAL FIGURE LEGENDS**

**Additional Figure 1**

*In vitro* nucleosome reconstitution on DNA with PRS-322. Nucleosomes were reconstituted *in vitro* using salt dialysis on a 220bp (A) or a 600bp (B) fragment of DNA harboring the 70bp PRS-322 element (highlighted in yellow). In both A and B the PRS-322 DNA and the flanking, non-highlighted DNA sequences are from the *C. elegans* genome, while the remaining sequences (highlighted in grey) are from the cloning vector. Below the full-length 220bp fragment sequence are the sequences from 27 aligned *in vitro* reconstituted nucleosome DNA cores derived from the 220bp fragment (A). Below the full-length 600bp fragment sequence are the sequences from 26 aligned *in vitro* reconstituted nucleosome DNA cores from the 600bp fragment (B). All sequence reads use the same highlighting scheme as the full-length DNA fragments from which they were derived. In both A and B, red arrows indicate nucleosomes that are potentially positioned due to end-bias.
